# Trade-offs in airway stability vs. hemodynamic safety: a retrospective study of alfentanil-sevoflurane-propofol anesthesia for tracheal intubation

**DOI:** 10.1016/j.clinsp.2025.100764

**Published:** 2025-09-20

**Authors:** Wanquan Ming, Yunfei Cao, Xianfei Xu, Miao Ding, Cheng Sheng

**Affiliations:** Department of Anesthesiology, Ningbo Beilun District People's Hospital, Ningbo, Zhejiang, China

**Keywords:** Airway management, Alfentanil, Bradycardia, Hypotension, Propofol, Sevoflurane, Tracheal intubation surgeries

## Abstract

**Background:**

Tracheal intubation surgeries are generally challenging procedures compared to other surgeries. Alfentanil is generally used to treat postoperative pain, but it has respiratory depression. The study examined four anesthetic techniques propofol/alfentanil, sevoflurane/alfentanil, propofol/sevoflurane, and propofol/sevoflurane/alfentanil to see if there is an association with a variety of anesthetic parameters for tracheal intubation surgery while maintaining spontaneous breathing.

**Methods:**

In retrospective analyses, patients received intravenous propofol and bolus alfentanil (PA cohort, *n* = 105) or exposed to sevoflurane gas and received bolus alfentanil (SA cohort, *n* = 107) or received intravenous propofol and exposed to sevoflurane gas (PS cohort, *n* = 109) or received intravenous propofol and bolus alfentanil and exposed to sevoflurane gas (SD cohort, *n* = 02).

**Results:**

Patients of the SD cohort achieved the best airway conditions, characterized by faster loss of eyelash reflex and quicker laryngeal mask insertion. However, patients of the SD cohort also exhibited worse hemodynamic stability, shorter operative times, higher postoperative complications, and prolonged hospital stays. Those results were followed for the patients of the PA, PS, and SA cohorts (*p* < 0.05 for all comparisons). Patients of the SD cohort required fewer numbers of postoperative administrations of a bolus dose of alfentanil than those of patients of the PS, PA, and SA cohorts (*p* < 0.05 for all comparisons).

**Conclusions:**

While the propofol-sevoflurane-alfentanil combination improved airway conditions, it caused universal hypotension and frequent bradycardia. Fixed-dose alfentanil (10 µg/kg) without titration may be unsafe in spontaneous breathing protocols due to respiratory depression.

## Highlights

• Propofol, sevoflurane, and alfentanil facilitate airway management.
• 6 % sevoflurane + 10 μg/kg of alfentanil can provide spontaneous breathing.
• Propofol + sevoflurane + alfentanil showed superior airway conditions.
• Propofol + sevoflurane + alfentanil had a hypotension risk and prolonged bradycardia.
• 10 µg/kg alfentanil without titration led to significant respiratory depression.

## Introduction

Tracheal intubation surgeries are generally challenging procedures compared to other surgeries.^1^ Direct laryngoscopy and video laryngoscopy are generally devices are used to perform it.^2^ A fiber bronchoscope is the device used in case of difficult intubation.^3^ Airway management is a generally critical issue during surgeries.^4^ A laryngeal mask airway is the preferred procedure for the preservation of spontaneous breathing in minor or moderate surgeries.^5^ These (minor or moderate) surgeries are generally managed with propofol.^6^ Moreover, propofol is superior to sevoflurane as an induction agent.^7^ Propofol provides larynx and pharynx relaxation to overcome coughing and gagging during laryngeal mask airway insertion that would lead to respiratory depression.^8^ However, sevoflurane is as good as propofol for anesthesia purposes^9^ and can maintain spontaneous breathing.^8^ Alfentanil is generally used to treat postoperative pain, but it has respiratory depression action.^10^ Spontaneous breathing anesthesia has fewer adverse effects than general anesthesia.^1^ There are several controversies regarding the actions of sevoflurane and propofol and their postoperative adverse effects because both have different mechanisms of action of anesthesia and different properties.^9^ Besides these (sevoflurane and propofol), a combination of alfentanil with sevoflurane has different actions and postoperative adverse effects than those of the combination of alfentanil with propofol.^11^ All agents (sevoflurane, propofol, and alfentanil) are currently used in anesthesia with different results as present strategies for anesthesia in tracheal intubation.

The authors have used these agents in different combinations for tracheal intubation surgery with preservation of spontaneous breathing. In addition, human factors, cognitive processes for decision-making, and strategies for application to preserve adequate alveolar oxygenation are taken into account for tracheal intubation surgery with preservation of spontaneous breathing.^12^ Goertzen C et al.^13^ have conducted a previous elegant review article and meta-analysis on this topic. They reported that the combination of vapor, midazolam, and a short-acting opioid is advantageous during apnea. However, they concluded that propofol combined with dexmedetomidine is the most effective anesthetic combination for minimizing adverse outcomes during laryngeal mask airway insertion. While the preservation of spontaneous breathing is crucial during tracheal intubation surgery, it is also necessary to evaluate other potential complications.

The objectives of the current retrospective medical records analyses of the study were to compare the pre-operative, perioperative, operative, and post-operative parameters of patients who received sevoflurane and propofol combination with alfentanil against those of patients who received sevoflurane and propofol combination without alfentanil, or sevoflurane with alfentanil, or propofol with alfentanil for tracheal intubation surgery while maintaining spontaneous breathing.

## Patients and methods

### Ethics approval and consent to participate

The protocol of the study was designed by the authors of the study, and these protocols were approved by the human ethics committee of the Ningbo Beilun District People’s Hospital and the Chinese Society of Anesthesiology. The approval number is 2024-50（K), dated December 14, 2020. The study follows the law of China and the 2008 Helsinki Declarations. Being a retrospective study, consent to participate of patients was waived by the human ethics committee of the Ningbo Beilun District People's Hospital.

### Design, setting, and period

A retrospective study of a collection of medical records of the Ningbo Beilun District People's Hospital, Ningbo, Zhejiang, China, from January 15, 2018, to 14 July 2022.

### Inclusion criteria

Patients with the American Society of Anesthesiologists (ASA) status 1 or 2, who underwent tracheal intubation surgery with preservation of spontaneous breathing, were included in the study. Laryngeal mask intubated patients with preserved spontaneous breathing, who are at risk of carbon dioxide accumulation and hypercapnia were selected for the study.

### Exclusion criteria

Patients who underwent tracheal intubation surgery underwent artificial breathing were excluded from the study. Patients with incomplete records (perioperative hemodynamic parameters and airway management) were also excluded from the study. Patients with hypotension (< 65 mmHg Systolic Blood Pressure [SBP]), hypertension (SBP > 90 mmHg and/ or Diastolic Blood Pressure [DBP] > 140 mmHg), and bradycardia (heart rate < 61 beats/min) before the operation were excluded from the study.

### Anesthesia methods

One hundred and five patients were subjected to administered intravenous 1.5 mg/kg propofol^10^ and received a bolus of 10 μg/kg of alfentanil^10^ (PA cohort). Hundred and seven patients were exposed to 6% sevoflurane gas in 60% nitrous oxide, 33% oxygen, and 1% air and maintained with 2% sevoflurane gas in 60% nitrous oxide, 33% oxygen, and 5% air for 10-minutes^11^ and received bolus 10 μg/kg of alfentanil (the institutional protocols do not allow titration of alfentanil) (SA cohort). One hundred and nine patients were administered intravenous 1.5 mg/kg propofol and exposed to 6% sevoflurane gas in 60% nitrous oxide, 33% oxygen, and 1% air and maintained with 2% sevoflurane gas in 60% nitrous oxide, 33% oxygen, and 5% air for 10 minutes (PS cohort). Hundred and two patients were administered intravenous 1.5 mg/kg propofol and exposed to 6% sevoflurane gas in 60% nitrous oxide, 33 % oxygen, and 1% air and maintained with 2% sevoflurane gas in 60% nitrous oxide, 33% oxygen, and 5% air for 10-minutes and received bolus 10 μg/kg of alfentanil (SD cohort). Hemodynamic parameters were monitored.^11^ Bolus alfentanil was administered immediately after the patients lost their eyelash reflex to avoid excitatory phenomena.^11^ The four anesthetic techniques selected for the subjects were decided after consulting anesthesiologists. Anesthetic dosing followed a strictly standardized regimen (e.g., 1.5 mg/kg propofol, 10 μg/kg alfentanil), was not adjusted based on patient-specific factors such as age, weight extremes, or comorbidities (fixed dosing; institutional protocol; prior studies demonstrate safety at 1 µg/kg in ASA 1–2 patients). Because of retrospective analyses, a power sample size calculation was not performed. The cohort recruited was opportunistic.

### Perioperative hemodynamic parameters evaluations

Perioperative hemodynamic parameters were evaluated with the help of a patient monitor device (CARESCAP B850 Monitor, GE Healthcare Pvt. Ltd., Beijing, China). SBP, DBP, Saturation of Peripheral Oxygen (SpO_2_) level, Mean Arterial Blood Pressure (MAP), and heart rates (beats/ min) were evaluated, and measures were taken to overcome abnormal results.

### Perioperative hemodynamic parameters management

A drop of 20 % or more in MAP from baseline (before induction) and/ or below 65 mmHg of DBP and/ or below 90 mmHg of SBP was considered hypotension. A total of 10 mg of bolus ephedrine was administered for hypotension. In the case of hypertension (high SBP and DBP), intravenous clonidine injection was given. If the heart rate falls less than 61 beats/min, then an atropine injection was administered to the patient to restore proper heart rhythm.^14^

### Perioperative airway management

A laryngeal mask airway was placed for spontaneous breathing by a consulting anesthesiologist. Laryngeal mask airway conditions were graded as per Table 1 on three-point scales. Parameters, for example, jaw opening, ease of laryngeal mask airway insertion, gagging, coughing, patient movements, and laryngospasm were used to grade laryngeal mask airway conditions. The overall laryngeal mask airway insertion conditions were evaluated based on the total score. Eighteen or more was considered excellent, a 16 to 17 total score was considered satisfactory, and a total score of 15 or below was considered poor.^11^ The time for loss of eyelash reflex and the time taken for laryngeal mask airway insertion were also noted.

**Table 1:** Laryngeal mask airway conditions grading on a three-point scale.

Condition(s)	Descriptions	Ordinal scale
Jaw opening	Full	3
	Partial	2
	Nil	1
Ease of laryngeal mask airway insertion	Easy	3
	Difficult	2
	Impossible	1
Gagging	Nil	3
	Slight	2
	Moderate	1
Coughing	Nil	3
	Slight	2
	Moderate	1
Patient movements	Nil	3
	Moderate	2
	Vigorous	1
Laryngospasm, airway obstruction	Nil	3
	Partial	2
	Total	1

An anesthesiologist performed grading. The higher the score better the condition(s).

Overall laryngeal mask airway insertion conditions: The total score ≥ 18: excellent, the total score: of 16 to 17: satisfactory, and the total score ≤ 15 : poor.

Through the endotracheal tube, the ventilator was connected to the trachea of patients. If the SpO_2_ level was less than 91 %, the ventilator was started through the jet and the configuration was as follows: driving pressure was 0.5–1.0 bar, FiO_2_ was 1.0, respiration rate was 60 breaths/min, and I:E ratio was 1:1. This was continued until the reach of SpO_2_ level more than 97 %. If there was no improvement in SpO_2_ level, even after connection of the jet to the trachea of the patient(s), high-frequency ventilation was made to get spontaneous breathing (1). A threshold of 91 % was chosen to minimize hypoxemia risk during spontaneous ventilation. According to this procedure, breathing of patients during the operation and in the Post-Operation Intensive Care Unit (PICU) was classified as per Table 2.

**Table 2:** Breathing of patients during operation and in post-operation intensive care unit.

Breathing type	Airway condition	SpO_2_ level
Spontaneous	Without the use of a ventilator	91–100
Spontaneous with mild frequency ventilator	Use of mild frequency ventilator	< 91 %
High-frequency ventilator	Use of High-frequency ventilator	< 80 %

SpO_2_, Saturation of Peripheral Oxygen.

### Operation time

Operation time was the time from entry into the operating room to entry into the PICU.

### Postoperative parameters

Length of stay in PICU, administration of a dose of alfentanil bolus in PICU and during ward stay, undesirable effects (nausea events, vomiting events, dyspnea events, dizziness events, numbness events, pain at injection sites, and more unwanted events), and post-operative hospital stay were evaluated from hospital records of patients.

### Statistical analysis

GraphPad InStat® 3 (Trial), GraphPad Software Inc., San Diego, CA, USA was used for statistical analysis purposes. Calculator Soup® (Quartile Calculator; https://www.calculatorsoup.com/calculators/statistics/quartile-calculator.php), CalculatorSoup, LLC., USA was preferred for interquartile range calculation and to find first Quartile (Q1), second Quartile (Q2) and third Quartile (Q3) values of non-normally distributed continuous variables and ordinal data set. Categorial, continuous normally distributed, and continuous non-normally distributed variables and ordinal data are demonstrated as frequencies with percentages in parentheses, mean ± Standard Deviation (SD), and medians with Q3–Q1 in parentheses, respectively. The Chi-Square test (χ^2^-test with or without Yate’s correction) or Fisher's exact test was preferred for the statistical analysis of categorical variables. One-way analysis of variance (ANOVA) was used for statistical analysis of continuous normally distributed variables or ordinal data. The Tukey-Kramer multiple comparisons test was used for *post hoc* analysis of continuous normally distributed variables or ordinal data to overcome type I error. The Kolmogorov-Smirnov test was used to check the normality of continuous variables. At least one column failed the normality test with *p* < 0.05, and Kruskal-Wallis’ test (nonparametric ANOVA) was preferred for statistical analysis of continuous non-normally distributed variables or ordinal data. Dunn's multiple comparisons test was used for *post hoc* analysis of continuous non-normally distributed variables or ordinal data to overcome type I errors. All results were considered significant if the p-value was less than 0.05.^15^

## Results

### Study population

**Fig. 1:**
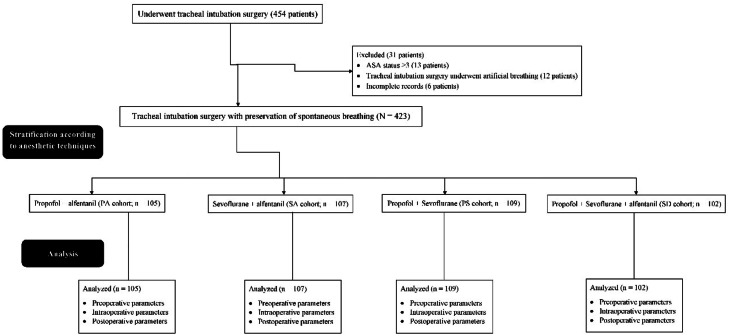
The study flow chart of retrospective medical records analyses. ASA, The American Society of Anesthesiologists.

From January 15, 2018, to 14 July 2022, a total of 454 patients underwent tracheal intubation surgery at the Ningbo Beilun District People's Hospital, Ningbo, Zhejiang, China, and the referring hospitals. Among them, 13 patients had ASA status 3 or higher, 12 patients underwent tracheal intubation surgery underwent artificial breathing, and complete records (more than 3 vital signs) of six patients were not available at the institutes. Therefore, the data of these patients (31 patients) were excluded from the study. Preoperative, perioperative, and postoperative parameters of a total of 423 patients were included in the analyses (these records were electronically stored). The study flow chart of retrospective medical records analysis is presented in Fig. 1.

### Preoperative parameters

Before the operation, age, ethnicity, body mass index, weight, gender, and ASA status were comparable among cohorts (*p* > 0.05 for all). In addition, hemodynamic parameters (SBP, DBP, Spo_2_, MAP, and heart rates) were comparable between cohorts before induction of anesthesia (*p* > 0.05 for all). The details of the demographic and clinical conditions of patients before the operation are presented in Table 3.

**Table 3:** Demographical and clinical conditions and hemodynamic parameters of patients before operation.

Parameters		Cohorts				Comparisons between cohorts		
		PA	SA	PS	SD			
Anesthetic technique		Propofol + alfentanil	Sevoflurane + alfentanil	Propofol + Sevoflurane	Propofol + Sevoflurane + alfentanil			
Numbers of patients		105	107	109	102	p-value	df	Test value
Age (years)		42 (45–40)	42 (44–41)	42 (43–40)	42 (43–40)	0.3319 (Kruskal-Wallis’ test)	N/A	3.416
Ethnicity	Han Chinese	96 (91)	99 (93)	101 (93)	92 (90)	0.9141 (χ^2^-test for Independence)	9	3.959
	Mongolian	7 (7)	7 (6)	7 (6)	8 (8)			
	Tibetan	1 (1)	1 (1)	1 (1)	2 (2)			
	Uyghur Muslims	1 (1)	0 (0)	0 (0)	0 (0)			
Body mass index (kg/m^2^)		25.7 (26.1–25)	25.9 (26.1–25)	25.9 (26.1–25.6)	25.5 (26.1–25)	0.1166 (Kruskal-Wallis’ test)	N/A	5.899
Weight (kg)		55 (57–52)	53 (56–51)	55 (56–51)	53 (55–51)	0.0713 (Kruskal-Wallis’ test)	N/A	7.02
Gender	Male	65 (62)	68 (64)	69 (63)	63 (62)	0.9898 (χ^2^-test for Independence)	3	0.1161
	Female	40 (38)	39 (36)	40 (37)	39 (38)			
ASA status	1	70 (67)	73 (43)	75 (69)	66 (65)	0.9235 (χ^2^-test for Independence)	3	0.479
	2	35 (33)	34 (57)	34 (31)	36 (35)			
SBP (mmHg)		125 (127–125)	125 (126–125)	126 (127–125)	125 (127–125)	0.1669 (Kruskal-Wallis’ test)	N/A	5.068
MAP (mmHg)		78 (79–77)	78 (79–74)	78 (80–77)	78 (80–75)	0.2418 (Kruskal-Wallis’ test)	N/A	4.188
DBP (mmHg)		87 (88–86)	86 (87–85)	86 (87–86)	86 (87–86)	0.5199 (Kruskal-Wallis’ test)	N/A	2.262
SpO_2_ level		99 (99–99)	99 (99–98)	99 (99–98)	99 (99–98)	0.9475 (Kruskal-Wallis’ test)	N/A	0.3645
Heart rates (beats/min)		88 (90–87.5)	88 (91–88)	88 (89–88)	88 (89–88)	0.1406 (Kruskal-Wallis’ test)	N/A	5.468

Categorial and continuous non-normal variables are demonstrated as frequencies with percentages in parenthesis and medians with Q3–Q1 in parenthesis, respectively. All results were considered significant if the p-value was less than 0.05.

ASA, The American Society of Anesthesiologists; df, Degree of freedom; N/A, Not Applicable; SBP, Systolic Blood Pressure; DBP, Diastolic Blood Pressure; MAP, Mean Arterial Blood Pressure; SpO_2_, Saturation of Peripheral Oxygen.

Test value (χ^2^-value for χ^2^-test, Kruskal-Wallis Statistic for Kruskal-Wallis’ test).

### Perioperative airway management

**Fig. 2:**
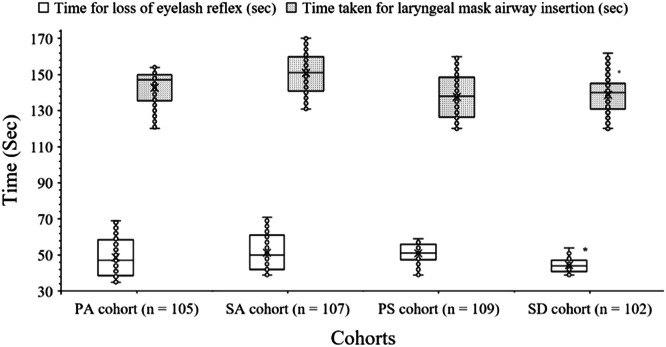
Perioperative airway parameters evaluations. The middle horizontal line in the box represents the median value. The box represents quartile values. Upper outlier: Maximum value, Lower outlier: Minimum value. *Fewer than those of the PA, PS, and SA cohorts (*p* < 0.05).

All patients had poor overall laryngeal mask airway insertion conditions. Laryngeal mask airway conditions (jaw opening, ease of laryngeal mask airway insertion, gagging, coughing, patient movements, laryngospasm, airway obstruction) grading were better in the patients of the SD cohort, followed by those of patients of the PS, PA, and SA cohorts (*p* < 0.05 for all, Kruskal-Wallis’ test/Dunn's multiple comparisons test). The details of perioperative airway management are reported in Table 4. The time for loss of eyelash reflex was 47 (58.5–38.5) sec per patient, 50 (61–42) sec per patient, 51 (56–47.5) sec per patient, and 44 (47–41) sec per patient for the PA, SA, PS, and SD cohorts, respectively. The time taken for laryngeal mask airway insertion was 147 (150–135.5) sec per patient, 151 (160–141) sec per patient, 138 (148.5–126.5) sec per patient, and 140 (145–131) sec per patient for the PA, SA, PS, and SD cohorts, respectively. The time for loss of eyelash reflex and the time taken for laryngeal mask airway insertion were fewer for patients of the SD cohort, followed by the PA, PS, and SA cohorts (*p* < 0.05 for all, Kruskal-Wallis’ test/Dunn's multiple comparisons test). The details of the time for loss of eyelash reflex and the time taken for laryngeal mask airway insertion are presented in Fig. 2.

**Table 4:** Perioperative laryngeal mask airway conditions grading evaluations.

Condition (s)	Cohorts									
	SD	PA			SA			PS		
Anesthetic technique	Propofol + Sevoflurane + alfentanil	Propofol + alfentanil			Sevoflurane + alfentanil			Propofol + Sevoflurane		
Numbers of patients	102	105	ap-value	aTest value	107	ap-value	aTest value	109	ap-value	aTest value
Jaw opening	2 (2–1)	1 (2–1)	<0.001	46.19	1 (2–1)	<0.05	46.19	2 (3–1)	>0.05	46.19
Ease of laryngeal mask airway insertion	2 (2–1)	1 (2–1)	>0.05	32.172	1 (2–1)	<0.05	32.172	2 (2–1)	>0.05	32.172
Gagging	2 (3–2)	1 (2–1)	<0.001	103.6	1 (2–1)	<0.001	103.6	2 (2–1)	<0.001	103.6
Coughing	2 (3–1)	1 (2–1)	<0.001	65.642	1 (2–1)	<0.001	65.642	2 (2–1)	<0.001	65.642
Patient movements	3 (3–2)	1 (1–1)	<0.001	169.35	1 (1–1)	<0.001	169.35	1 (2–1)	<0.001	169.35
Laryngospasm, airway obstruction	3 (3–2)	1 (1–1)	<0.001	193.34	1 (1–1)	<0.001	193.34	1 (2–1)	<0.001	193.34
Overall laryngeal mask airway insertion conditions	13 (14–12)	7 (8.5–7)	<0.001	237.82	8 (9–7)	<0.001	237.82	10 (11–9)	<0.001	237.82

Variables are demonstrated as medians with Q3–Q1 in parenthesis, respectively.

Kruskal-Wallis’ test was used for statistical analysis of non-normal continuous variables. Dann’s multiple comparison test was used for *post hoc* analysis of non-normal continuous variables.

All results were considered significant if the p-value was less than 0.05.

a Concerning the SD cohort. An anesthesiologist performed grading. The higher the score better the condition(s). Overall laryngeal mask airway insertion conditions: The total score ≥18: excellent, the total score: of 16 to 17: satisfactory, and the total score ≤15 poor. Test value (Kruskal-Wallis’ Statistic for Kruskal-Wallis’ test). N/A, Not Applicable; df, degree of freedom; CI, Confidence Interval (using the approximation of Katz.) for categorical variables.

### Perioperative hemodynamic parameters

After induction SBP, DBP, and MAP values decreased in all cohorts. However, SBP, DBP, and MAP values decreased at their maximum in the SD cohort, followed by those of patients of the PS cohort, the SA cohort, and the PA cohort. None of the patients reported hypertension during the operation. The total number of patients with reported hypotension during operation and the numbers of 10 mg bolus ephedrine during operation/patient were higher in the SD cohort than those of the PS cohort, the SA cohort, and the PA cohort. In addition, the total number of patients with reported hypotension during operation and the number of 10 mg bolus ephedrine administration during operation per patient were higher in the PS cohort than those of the SA cohort and the PA cohort. The details of perioperative systolic and diastolic blood pressures and mean arterial blood pressure evaluations during the operation are reported in Table 5.

**Table 5:** Perioperative systolic and diastolic blood pressures and mean arterial blood pressure evaluations.

Time level respect to induction for different hemodynamic parameters	Cohorts												
	SD	PA				SA				PS			
Anesthetic technique	Propofol + Sevoflurane + alfentanil	Propofol + alfentanil				Sevoflurane + alfentanil				Propofol + Sevoflurane			
Numbers of patients	102	105	ap-value	aTest value	a95 % CI	107	ap-value	aTest value	a95 % CI	109	ap-value	aTest value	a95 % CI
SBP (mmHg)													
Before induction	125 (127–125)	125 (127–125)	>0.05	5.068	N/A	125 (126–125)	>0.05	5.068	N/A	126 (127–125)	>0.05	5.068	N/A
1 min after induction	116 (119–115)	116 (118–115)	>0.05	23.198	N/A	118 (119–115)	>0.05	23.198	N/A	116 (119–115)	>0.05	23.198	N/A
Between 2 to 10 min after induction	105 (106–104)	108 (111–106)	<0.001	164.34	N/A	111 (113–109)	<0.001	164.34	N/A	105 (106–104)	>0.05	164.34	N/A
Between 10 to 30 min after induction	95 (96—94)	102 (104–101)	<0.001	263.53	N/A	107 (108–103)	<0.001	263.53	N/A	100 (102–97)	<0.001	263.53	N/A
After 30 min till operation continued	84 (85–84)	92 (95–91.5)	<0.001	289.36	N/A	96 (98–93)	<0.001	289.36	N/A	89 (91–86)	<0.001	289.36	N/A
DBP (mmHg)													
Before induction	86 (87–86)	87 (88–86)	>0.05	2.262	N/A	86 (87–85)	>0.05	2.262	N/A	86 (87–86)	>0.05	2.262	N/A
1 min after induction	83 (84–82)	85 (85–84.5)	<0.001	90.723	N/A	85 (85–84)	<0.001	90.723	N/A	83 (84–83)	>0.05	90.723	N/A
Between 2 to 10 min after induction	79 (80–78)	82 (81–82)	<0.001	125.82	N/A	81 (82–79)	<0.001	125.82	N/A	79 (80.5–79)	<0.01	125.82	N/A
Between 10 to 30 min after induction	75 (75–73)	78 (79–77)	<0.001	171.66	N/A	77 (79–75)	<0.001	171.66	N/A	75 (77–75)	<0.001	171.66	N/A
After 30 min till operation continued	69 (70–68)	74 (75–72)	<0.001	235.78	N/A	74 (75–72)	<0.001	235.78	N/A	70 (69–72)	<0.001	235.78	N/A
MAP (mmHg)													
Before induction	78 (80–75)	78 (79–77)	>0.05	4.188	N/A	78 (79–74)	>0.05	4.188	N/A	78 (80–77)	>0.05	4.188	N/A
1 min after induction	74 (75–71)	76 (76.5–75)	<0.001	37.063	N/A	75 (76–72)	<0.01	37.063	N/A	76 (78–75)	<0.001	37.063	N/A
Between 2 to 10 min after induction	69 (70–66)	72 (73–71)	<0.001	75.425	N/A	72 (73–68)	<0.001	75.425	N/A	72 (73–70)	<0.001	75.425	N/A
Between 10 to 30 min after induction	65 (66–62)	69 (70–68)	<0.001	98.658	N/A	68 (70–65)	<0.001	98.658	N/A	67 (69–65.5)	<0.001	98.658	N/A
After 30 min till operation continued	63 (64–60)	66 (67–64)	<0.001	73.745	N/A	65 (67–62)	<0.001	73.745	N/A	64 (65–62)	<0.01	73.745	N/A
Number of patients with hypotension													
Before induction	0 (0)	0 (0)	N/A	N/A	N/A	0 (0)	N/A	N/A	N/A	0 (0)	N/A	N/A	N/A
1 min after induction	0 (0)	0 (0)	N/A	N/A	N/A	0 (0)	N/A	N/A	N/A	0 (0)	N/A	N/A	N/A
Between 2 to 10 min after induction	0 (0)	0 (0)	N/A	N/A	N/A	0 (0)	N/A	N/A	N/A	0 (0)	N/A	N/A	N/A
Between 10 to 30 min after induction	11 (11)	0 (0)	0.0003	2.154	1.853 to 2.503	1 (1)	0.0022	1.984	1.580 to 2.492	1 (1)	0.002	2.005	1.596 to 2.518
After 30 min till operation continued	102 (100)	9 (9)	<0.0001	Infinity	-Infinity to Infinity	22 (21)	<0.0001	Infinity	-Infinity to Infinity	89 (82)	<0.0001	Infinity	-Infinity to Infinity
The total number of patients who reported hypotension during any stage of operation	102 (100)	9 (9)	<0.0001	Infinity	-Infinity to Infinity	23 (21)	<0.0001	Infinity	-Infinity to Infinity	90 (83)	<0.0001	Infinity	-Infinity to Infinity
Numbers of 10 mg of bolus ephedrine during operation/patient	1 (2–1)	0 (0–0)	<0.001	229.36	N/A	0 (0–0)	<0.001	229.36	N/A	1 (2–0)	<0.001	229.36	N/A

Categorial and continuous non-normal variables are demonstrated as frequencies with percentages in parenthesis and medians with Q3–Q1 in parenthesis, respectively.

All results were considered significant if the p-value was <0.05.

SBP, Systolic Blood Pressure; DBP, Diastolic Blood Pressure; MAP, Mean Arterial Blood Pressure; N/A, Not Applicable; CI, Confidence Interval (using the approximation of Katz.) for categorical variables.

The average of five measures was taken for analysis. The value was reading before administration of 10 mg of bolus ephedrine (when applicable).

Kruskal-Wallis’ test was used for statistical analysis of non-normal continuous variables. Dann’s multiple comparison test was used for *post hoc* analysis of non-normal continuous variables.

Fisher's exact test was used for statistical analysis of categorial variables.

a Concerning the SD cohort. Test value (Kruskal-Wallis Statistic for Kruskal-Wallis’ test; Relative risk for Fisher's exact test). Hypotension: A drop of ≥20 % in MAP from baseline (before induction) and/ or < 65 mmHg of DBP and/ or < 90 mmHg of SBP.

After induction, SpO_2_ levels decreased in all patients. Several patients with decreased SpO_2_ levels and fewer patients with spontaneous breathing were reported in patients of the SD and patients of the PS cohorts, followed by patients of the SA cohort and patients of the PA cohort. The details of perioperative saturation of peripheral oxygen evaluations during operation are reported in Table 6. Patients with bradycardia (heart rate < 60 beats/minute) were reported in the SD and the PS cohorts, followed by the SA cohort and the PA cohort. Requirements of atropine injection were mainly in the SD and PS cohorts. The details of perioperative heart rate evaluations are reported in Table 7.

**Table 6:** Perioperative saturation of peripheral oxygen evaluations.

Time level respect to induction for different breathing type conditions		Cohorts												
		SD	PA				SA				PS			
Anesthetic technique		Propofol + Sevoflurane + alfentanil	Propofol + alfentanil				Sevoflurane + alfentanil				Propofol + Sevoflurane			
Numbers of patients		102	105	ap-value	aTest value	a95 % CI	107	ap-value	aTest value	a95 % CI	109	ap-value	aTest value	a95 % CI
Saturation of peripheral oxygen														
Before induction		99 (99–98)	99 (99–99)	>0.05	0.3645	N/A	99 (99–98)	>0.05	0.3645	N/A	99 (99–98)	>0.05	0.3645	N/A
1 min after induction		92 (93–92)	95 (96–95)	<0.001	239.78	N/A	95 (95–94)	<0.001	239.78	N/A	94 (95–93)	<0.001	239.78	N/A
Between 2 to 10 min after induction		90 (91–90)	92 (92–91.5)	<0.001	111.1	N/A	92 (92–91)	<0.001	111.1	N/A	91 (92–90)	<0.01	111.1	N/A
Between 10 to 30 min after induction		88 (91–85)	91 (91–90)	<0.001	56.234	N/A	91 (91–90)	<0.001	56.234	N/A	90 (91–86)	>0.05	56.234	N/A
After 30 min till operation continued		81 (84–77)	83 (85–83)	<0.001	54.424	N/A	84 (88–83)	<0.001	54.424	N/A	83 (84–81)	<0.05	54.424	N/A
Breathing of patients during operation														
Before induction	Spontaneous	102 (100)	105 (100)	N/A	N/A	N/A	107 (100)	N/A	N/A	N/A	109 (100)	N/A	N/A	N/A
	Spontaneous with mild frequency ventilator	0 (0)	0 (0)				0 (0)				0 (0)			
	High-frequency ventilator	0 (0)	0 (0)				0 (0)				0 (0)			
1 min after induction	Spontaneous	102 (100)	105 (100)	N/A	N/A	N/A	107 (100)	N/A	N/A	N/A	109 (100)	N/A	N/A	N/A
	Spontaneous with mild frequency ventilator	0 (0)	0 (0)				0 (0)				0 (0)			
	High-frequency ventilator	0 (0)	0 (0)				0 (0)				0 (0)			
Between 2 to 10 min after induction	Spontaneous	47 (46)	91 (87)	<0.0001	0.4273	0.3291 to 0.5547	84 (79)	<0.0001	22.107 (df = 1)	0.3883 to 0.6667	72 (66)	0.0053	7.758 (df = 1)	0.5000 to 0.8729
	Spontaneous with mild frequency ventilator	55 (54)	14 (13)				23 (21)				37 (34)			
	High-frequency ventilator	0 (0)	0 (0)				0 (0)				0 (0)			
Between 10 to 30 min after induction	Spontaneous	32 (31)	63 (60)	<0.0001	15.942 (df = 1)	0.3927 to 0.7397	59 (55)	0.0009	11.053 (df = 1)	0.4319 to 0.8135	48 (44)	0.0793	3.072 (df = 1)	0.5477 to 1.023
	Spontaneous with mild frequency ventilator	70 (69)	42 (40)				48 (45)				61 (56)			
	High-frequency ventilator	0 (0)	0 (0)				0 (0)				0 (0)			
After 30 min till operation continued	Spontaneous	0 (0)	17 (16)	<0.0001	30.409 (df = 2)	N/A	25 (23)	<0.0001	33.991 (df = 2)	N/A	0 (0)	0.1232	2.376 (df = 1)	0.5807 to 1.024
	Spontaneous with mild frequency ventilator	70 (69)	79 (75)				70 (66)				86 (79)			
	High-frequency ventilator	32 (31)	9 (9)				12 (11)				23 (21)			

Categorial and continuous non-normal variables are demonstrated as frequencies with percentages in parenthesis and medians with Q3–Q1 in parenthesis, respectively.

Kruskal-Wallis’ test was used for statistical analysis of non-normal continuous variables. Dann’s multiple comparison test was used for *post hoc* analysis of non-normal continuous variables.

Fisher's exact test or χ^2^-test (with Yates correction) of χ^2^-test (for Independence) was used for statistical analysis of categorial variables.

All results were considered significant if the p-value was less than 0.05.

a Concerning the SD cohort. The average of five measures was taken for analysis. The value was read before the ventilator was started through jet or high-frequency ventilation or saturated condition. Test value (Kruskal-Wallis Statistic for Kruskal-Wallis’ test; Relative risk for Fisher's exact test, χ^2^-value (with Yates correction) for χ^2^-test). N/A, Not Applicable; CI, Confidence Interval (using the approximation of Katz.) for categorical variables; df, Degree of freedom.

**Table 7:** Perioperative heart rate evaluations.

Different conditions respect to induction of anesthesia	Cohorts														
	SD	PA				SA					PS				
Anesthetic technique	Propofol + Sevoflurane + alfentanil	Propofol + alfentanil				Sevoflurane + alfentanil					Propofol + Sevoflurane				
Numbers of patients	102	105	ap-value	aTest value	a95 % CI	107	ap-value	df	aTest value	a95 % CI	109	ap-value	df	aTest value	a95 % CI
Before induction	88 (89–88)	88 (90–87.5)	>0.05	5.468	N/A	88 (91–88)	>0.05	N/A	5.468	N/A	88 (89–88)	>0.05	N/A	5.468	N/A
1 min after induction	80 (80–78)	79 (81–78)	>0.05	4.978	N/A	80 (81–78)	>0.05	N/A	4.978	N/A	80 (81–79)	>0.05	N/A	4.978	N/A
Between 2 to 10 min after induction	71.5 (73–69)	72 (73–70)	<0.05	10.505	N/A	72 (73–70)	>0.05	N/A	10.505	N/A	72 (73–71)	<0.05	N/A	10.505	N/A
Between 10 to 30 min after induction	60 (70–60)	68 (70–67)	<0.001	26.77	N/A	69 (70–67)	<0.001	N/A	26.77	N/A	68 (69–66)	<0.01	N/A	26.77	N/A
After 30 min till operation continued	62 (64–60)	64 (64–63)	<0.001	45.495	N/A	64 (64–62)	<0.001	N/A	45.495	N/A	62 (64–60)	>0.05	N/A	45.495	N/A
Number of patients with bradycardia															
Before induction	0 (0)	0 (0)	N/A	N/A	N/A	0 (0)	N/A	N/A	N/A	N/A	0 (0)	N/A	N/A	N/A	N/A
1 min after induction	0 (0)	0 (0)	N/A	N/A	N/A	0 (0)	N/A	N/A	N/A	N/A	0 (0)	N/A	N/A	N/A	N/A
Between 2 to 10 min after induction	0 (0)	0 (0)	N/A	N/A	N/A	0 (0)	N/A	N/A	N/A	N/A	0 (0)	N/A	N/A	N/A	N/A
Between 10 to 30 min after induction	53 (52)	0 (0)	<0.0001	3.143	2.494 to 3.961	0 (0)	<0.0001	N/A	3.143	2.494 to 3.961	10 (9)	<0.0001	N/A	2.541	1.973 to 3.272
After 30 min till operation continued	46 (45)	2 (2)	<0.0001	2.721	2.186 to 3.387	18 (17)	<0.0001	1	18.344	1.440 to 2.404	42 (39)	0.4083	1	0.6838	0.8695 to 1.516

Categorial and continuous non-normal variables are demonstrated as frequencies with percentages in parenthesis and medians with Q3–Q1 in parenthesis, respectively.

Kruskal-Wallis’ test was used for statistical analysis of non-normal continuous variables. Dann’s multiple comparison test was used for *post hoc* analysis of non-normal continuous variables.

Fisher's exact test or χ^2^-test was used for statistical analysis of categorial variables.

All results were considered significant if the p-value was less than 0.05.

a Concerning the SD cohort. The average of five measures was taken for analysis. The value was reading before the administration of atropine injection. Test value (Kruskal-Wallis Statistic for Kruskal-Wallis’ test; Relative risk for Fisher's exact test; χ^2^-value for χ^2^-test). N/A, Not Applicable; df, Degree of freedom; CI, Confidence Interval (using the approximation of Katz.) for categorical variables.

### Operation time

**Fig. 3:**
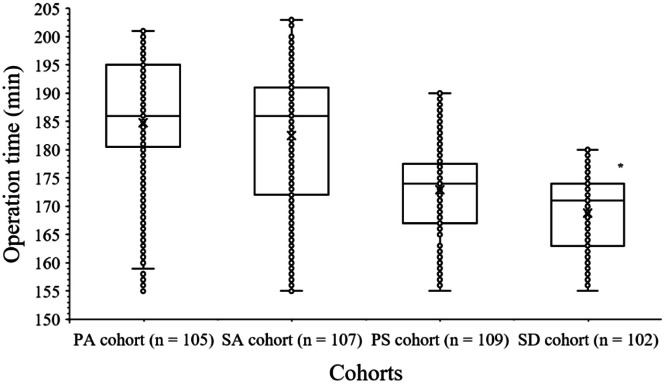
Operation time evaluation. Operation time: Time from entry into the operation room to entry into the post-surgical intensive care unit stay. The middle horizontal line in the box represents the median value. The box represents quartile values. Upper outlier: Maximum value, Lower outlier: Minimum value. *Fewer than those of the PA, PS, and SA cohorts (*p* < 0.05).

Patients of the SD cohort have less operative time than of patients of the PS, PA, and SA. The details of operative time are presented in Fig. 3.

### Postoperative parameters

There were no symptoms of cough and hoarseness in any of the patients in the PICU and during their ward stays. In addition, there were no symptoms of renal failure, bronchitis, asthma, etc. in the follow-up period. The length of stay in the PICU of patients was comparable among cohorts. Patients of the SD cohort required fewer numbers of administration of doses of alfentanil bolus than those of patients of the PS, PA, and SA cohorts (*p* < 0.05 for all, Kruskal-Wallis’ test/Dunn's multiple comparisons test). Twelve (12 %), 13 (12 %), 12 (11 %), and 17 (16 %) patients from the PA, SA, PS, and SD cohorts, respectively, faced nausea events. Three (3 %), 5 (5 %), 5 (5 %), and 5 (5 %) from the PA, SA, PS, and SD cohorts, respectively, faced vomiting events. Post-operative hospital stays of patients were fewer for patients of the PA (*p* < 0.001, Kruskal-Wallis’ test/Dunn's multiple comparisons test) and SA cohorts (*p* < 0.05, Kruskal-Wallis’ test/Dunn's multiple comparisons test) than those for patients of the SD cohort. The common undesirable effect in the SD, PA, and PS cohorts was pain at the injection site. The details of postoperative parameters in the PICU and during ward stays are reported in Table 8.

**Table 8:** Postoperative parameters in the post-surgical intensive care unit and during ward stay.

Event	Cohorts												
	SD	PA				SA				PS			
Anesthetic technique	Propofol + Sevoflurane + alfentanil	Propofol + alfentanil				Sevoflurane + alfentanil				Propofol + Sevoflurane			
Numbers of patients	102	105	ap-value	aTest value	a95 % CI	107	ap-value	aTest value	a95 % CI	109	ap-value	aTest value	a95 % CI
Operation time (min)	171 (174–163)	186 (195–180.5)	<0.001	118.69	N/A	186 (191–172)	<0.001	118.69	N/A	174 (177.5–167)	>0.05	118.69	N/A
Length of stays in PICU (hours)	23 (25–22)	24 (25–22)	>0.05	0.3958	N/A	24 (25–22)	>0.05	0.3958	N/A	23 (25–22)	>0.05	0.3958	N/A
Administration of dose of alfentanil bolus	4 (4–4)	5 (6–5)	<0.001	85.754	N/A	5 (6–4)	<0.001	85.754	N/A	4 (5–3)	>0.05	85.754	N/A
Undesirable effects													
Number of patients with nausea	12 (12)	13 (12)	0.999	0.9707	0.6291 to 1.498	12 (11)	0.999	1.028	0.6708 to 1.575	17 (16)	0.4328	0.8368	0.5296 to 1.322
Number of patients with vomiting	3 (3)	5 (5)	0.7214	0.7538	0.3047 to 1.865	5 (5)	0.7218	0.7614	0.3078 to 1.883	5 (5)	0.7225	0.7689	0.3108 to 1.902
Number of patients with dyspnea	1 (1)	0 (0)	0.4928	2.04	1.774 to 2.344	1 (1)	0.999	1.025	0.2544 to 4.128	1 (1)	0.999	1.035	0.2569 to 4.168
Number of patients with dizziness	1 (1)	2 (2)	0.999	0.6733	0.1350 to 3.357	1 (1)	0.999	1.025	0.2544 to 4.128	1 (1)	0.999	1.035	0.2569 to 4.168
Number of patients with numbness	3 (3)	2 (2)	0.6799	1.224	0.5902 to 2.539	3 (3)	0.999	1.025	0.4549 to 2.311	4 (4)	0.999	0.8831	0.3710 to 2.102
Numbers of patients with pain at the injection site	9 (9)	9 (9)	0.999	1.016	0.6261 to 1.649	1 (1)	0.0086	1.926	1.493 to 2.484	9 (9)	0.999	1.038	0.6391 to 1.685
Post-operative hospital stays (days)	4 (5–3)	3 (4–3)	<0.001	27.66	N/A	4 (4–3)	<0.05	27.66	N/A	4 (4.5–3)	>0.05	27.66	N/A

Categorial and continuous non-normal variables are demonstrated as frequencies with percentages in parenthesis and medians with Q3–Q1 in parenthesis, respectively.

Kruskal-Wallis’ test was used for statistical analysis of non-normal continuous variables. Dann’s multiple comparison test was used for *post hoc* analysis of non-normal continuous variables.

Fisher's exact test was used for statistical analysis of categorial variables.

All results were considered significant if the *p*-value was less than 0.05.

a Concerning the SD cohort. Test value (Kruskal-Wallis Statistic for Kruskal-Wallis’ test; Relative risk for Fisher's exact test). N/A, Not Applicable; CI, Confidence Interval (using the approximation of Katz.) for categorical variables; PICU, Post-surgical Intensive Care Unit.

## Discussions

The study reported that airway management was best in patients of the SD cohort, followed by those in patients of the PS and the PA cohorts. The results of the airway management of the current study are consistent with those of a prospective, randomized parallel study.^11^ Propofol,^16^ sevoflurane,^11^ and alfentanil^17^ all have strong depressant effects on the upper airway reflex. In addition, propofol and alfentanil combination^18^ and sevoflurane and alfentanil combination^11^ have synergistic depressant effects on the upper airway reflex. Propofol, sevoflurane, and alfentanil were all reported to facilitate airway management during surgeries. Benefits of the SD cohort regimen (propofol/sevoflurane/alfentanil) are required to evaluate adequately addressing its risks (e.g., 100 % hypotension rate) in future studies.

Patients of the SD and PS cohorts had higher numbers of postoperative complications. The results of the postoperative complications of the current study are consistent with those of a prospective, randomized parallel study^11^ and a systematic review and meta-analysis.^9^ Propofol is responsible for nausea, vomiting, and pain at the injection site.^11^ Alfentanil also causes nausea, vomiting, and dizziness.^19^ Propofol and alfentanil both reported postoperative complications in the post-surgical intensive care unit and during ward stay.

Hemodynamic Trade-offs: SD cohort (propofol + sevoflurane + alfentanil) showed superior airway conditions but 100 % hypotension risk and prolonged bradycardia. Fixed dose alfentanil (10 µg/kg) without titration led to significant respiratory depression (SpO_2_ < 91 % in 69 % of SD cohort). Propofol and alfentanil both reported hemodynamic instabilities (e.g., hypotension, bradycardia)^18^ and respiratory instabilities (e.g., dyspnea).^6^ In addition, sevoflurane and alfentanil has a cardiac depressant effect^11^ and alfentanil has a respiratory depressant action.^10^ The Propofol, sevoflurane, and alfentanil combination regimen should be avoided in hemodynamically unstable patients; consider lower alfentanil doses or alternative regimens.

Spontaneous breathing was reported in the maximum number of patients in the SA cohort, followed by those in patients of the PA, PS, and SD cohorts. The results of the spontaneous breathing of the current study are consistent with those of the prospective, randomized parallel study.^11^ Six percent sevoflurane gas with a bolus of 10 μg/kg of alfentanil provides spontaneous breathing.^11^ However, propofol depresses the skeletal muscle of the respiratory system.^16^ Six percent sevoflurane gas with a bolus of 10 μg/kg of alfentanil can provide spontaneous breathing.

The preoperative backgrounds appear to be too uniform. Is it possible that the preoperative backgrounds are overly consistent. The average heart rate seems high for individuals in their 40 s. Additionally, the age of 40 years is relatively young, making it inappropriate to generalize these results. In recent years, there has been a trend towards an increasing number of surgeries in elderly patients. As noted in previous studies,^20^ the study must consider patients aged 60 and above. Furthermore, these results of the current study may not be applicable to patient groups requiring preservation of spontaneous breathing more than those in this study. Particularly regarding the primary outcome measure of respiration, elderly patients are more likely to experience respiratory depression. The possible justification for the same is that in the institute were not performing surgeries on patients aged 60 and above.

The study provided reasons as to why findings are looking at anesthetic parameters of four anesthetic techniques and how they may affect their anesthetic management. However, there are some limitations of the study, for example, retrospective analyses and a lack of a dynamic study or randomization. Long-term outcomes are not evaluated. The potential for selection bias. In addition, there is no distinction based on the type of surgery, and this explains the variability in the results. Inherent biases (selection, unmeasured confounders) limit causal inferences. For example, the choice of anesthetic regimen may reflect patient-specific factors not accounted for. The manuscript does not specify the types of tracheal intubation surgeries included. Variability in surgical complexity could skew outcomes (e.g., longer operative times in certain procedures). Postoperative complications (e.g., nausea, hypotension) are reported as frequencies but lack severity grading or time-to-event analysis. Single-center study (China) with homogeneous cohorts (ASA 1–2, Han Chinese predominance). Extrapolation to higher-risk patients (ASA ≥3) or other populations is uncertain. The study provides something about alfentanil which would make it different than other commonly used opioids for the induction of anesthesia (fentanyl, remifentanil, sufentanil). No multivariate analysis (e.g., logistic regression) for age/BMI (limitations of the study). Single-center retrospective design limits generalizability; prospective randomized controlled trials are needed to validate findings. Non-random allocation may have skewed outcomes due to unmeasured confounders like surgeon preference or patient comorbidities.

This study is significant for its potential to refine anesthetic strategies in tracheal intubation surgeries, underscoring the importance of meticulous anesthetic selection to optimize both airway conditions and patient recovery.

## Conclusions

While the propofol-sevoflurane-alfentanil combination improved airway conditions, it caused universal hypotension and frequent bradycardia, necessitating vigilant hemodynamic monitoring. Fixed-dose alfentanil (10 µg/kg) without titration may be unsafe in spontaneous breathing protocols due to respiratory depression. Future prospective studies should optimize dosing and assess outcomes in high-risk populations (ASA ≥ 3).

## Abbreviations

ASA, The American Society of Anesthesiologists; PA cohort: Patients were subjected to administered intravenous 1.5 mg/kg propofol and received bolus 10 μg/kg of alfentanil; SA cohort: Patients were exposed to 6 % sevoflurane gas in 60 % nitrous oxide, 33 % oxygen, and 1 % air and maintained with 2 % sevoflurane gas in 60 % nitrous oxide, 33 % oxygen, and 5 % air for 10-minutes and received bolus 10 μg/kg of alfentanil; PS cohort: Patients administered intravenous 1.5 mg/kg propofol and exposed to 6 % sevoflurane gas in 60 % nitrous oxide, 33 % oxygen, and 1 % air and maintained with 2 % sevoflurane gas in 60 % nitrous oxide, 33 % oxygen, and 5 % air for 10-minutes; SD cohort: Patients administered intravenous 1.5 mg/kg propofol and exposed to 6 % sevoflurane gas in 60 % nitrous oxide, 33 % oxygen, and 1 % air and maintained with 2 % sevoflurane gas in 60 % nitrous oxide, 33 % oxygen, and 5 % air for 10-minutes and received bolus 10 μg/kg of alfentanil; SpO_2_, Saturation of Peripheral Oxygen; SBP, Systolic Blood Pressure; DBP, Diastolic Blood Pressure; PICU, Post-surgical Intensive Care Unit stay; SD, Standard Deviation; χ^2^-test, Chi-Square test; MAP, Mean Arterial Blood Pressure; ANOVA, Analysis of Variance; Q1, First Quartile; Q2, Second Quartile; Q3, Third Quartile.

## Declaration

All the data and related metadata underlying the reported findings are already provided as part of the submitted article. There are no Supplementary Files (Supplementary Tables, Supplementary Figures, and others) referred to in the manuscript. Therefore, there is nothing to deposit in appropriate public data repositories.

## Authors’ contributions

All authors have read and approved the final work publication. WM was the project administrator and contributed to the supervision, funding acquisition, resources, validation, and literature review of the study. YC contributed to software, methodology, funding acquisition, conceptualization, supervision, and literature review of the study. XX contributed to the investigation, methodology, funding acquisition, literature review, supervision, and software of the study. MD contributed to the methodology, resources, funding acquisition, validation, supervision, and literature review of the study. CS contributed to the methodology, formal analysis, data curation, supervision, funding acquisition, and literature review of the study. All authors contributed to the drafting and editing of the manuscript for intellectual content. All authors agree to be accountable for all aspects of the work, ensuring its integrity and accuracy.

## Funding support

This study was supported by the Medical and Health Science and Technology Project of Zhejiang Province, China (No.2024XY036).

## Availability of data and materials

The datasets used and analyzed during the current study are available from the corresponding author upon reasonable request.

## Declaration of competing interest

The authors declare that they have no conflicts of interest or any other competing interests regarding the results and/or discussion reported in the research.
